# Successful osimertinib retreatment after extremely early onset severe pneumonitis in first‐line treatment of lung adenocarcinoma

**DOI:** 10.1111/1759-7714.13565

**Published:** 2020-07-15

**Authors:** June Hong Ahn

**Affiliations:** ^1^ Division of Pulmonology and Allergy, Department of Internal Medicine, College of Medicine, Yeungnam University and Regional Center for Respiratory Diseases Yeungnam University Medical Center Daegu South Korea

**Keywords:** Lung cancer, osimertinib, pneumonitis, retreatment

## Abstract

Drug‐induced pneumonitis is rare, and can result in death. Here, we present a report of a patient with adenocarcinoma harboring EGFR exon 19 deletion mutation treated with osimertinib as first‐line treatment. After six days of treatment, extremely early onset severe pneumonitis was diagnosed. Discontinuation of osimertinib as well as administration of corticosteroid, and retreatment with osimertinib were successful. This case report highlights that extremely early onset severe pneumonitis can occur after osimertinib administration, and retreatment of osimertinib may be a useful treatment option after resolution of pneumonitis.

## Graphical abstract

Osimertinib can be associated with pneumonitis. This case highlights extremely early onset with severe pneumonitis. In such case, discontinuation of osimertinib as well as administration of corticosteroid, and retreatment with osimertinib carefully may be a fine treatment option especially when patients have brain metastasis.

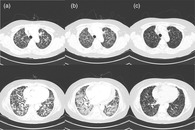

## Introduction

Osimertinib is a third‐generation, irreversible epidermal growth factor receptor tyrosine kinase inhibitor (EGFR‐TKI), which shows great efficacy in first‐line treatment of EGFR mutation‐positive advanced non‐small cell lung cancer (NSCLC).^1^ Similar to other EGFR‐TKIs, osimertinib‐induced pneumonitis has been reported at rates of 2%–4% across clinical trials, and drug‐induced pneumonitis can result in death.^2^ To date, and to the best of our knowledge, there have been no reports of osimertinib‐induced pneumonitis occurring within one week of treatment. Discontinuation of the drug is recommended in cases of severe pneumonitis. Here, we report a case of successful retreatment after extremely early onset severe pneumonitis in first‐line treatment of lung adenocarcinoma.

## Case report

**Figure 1:**
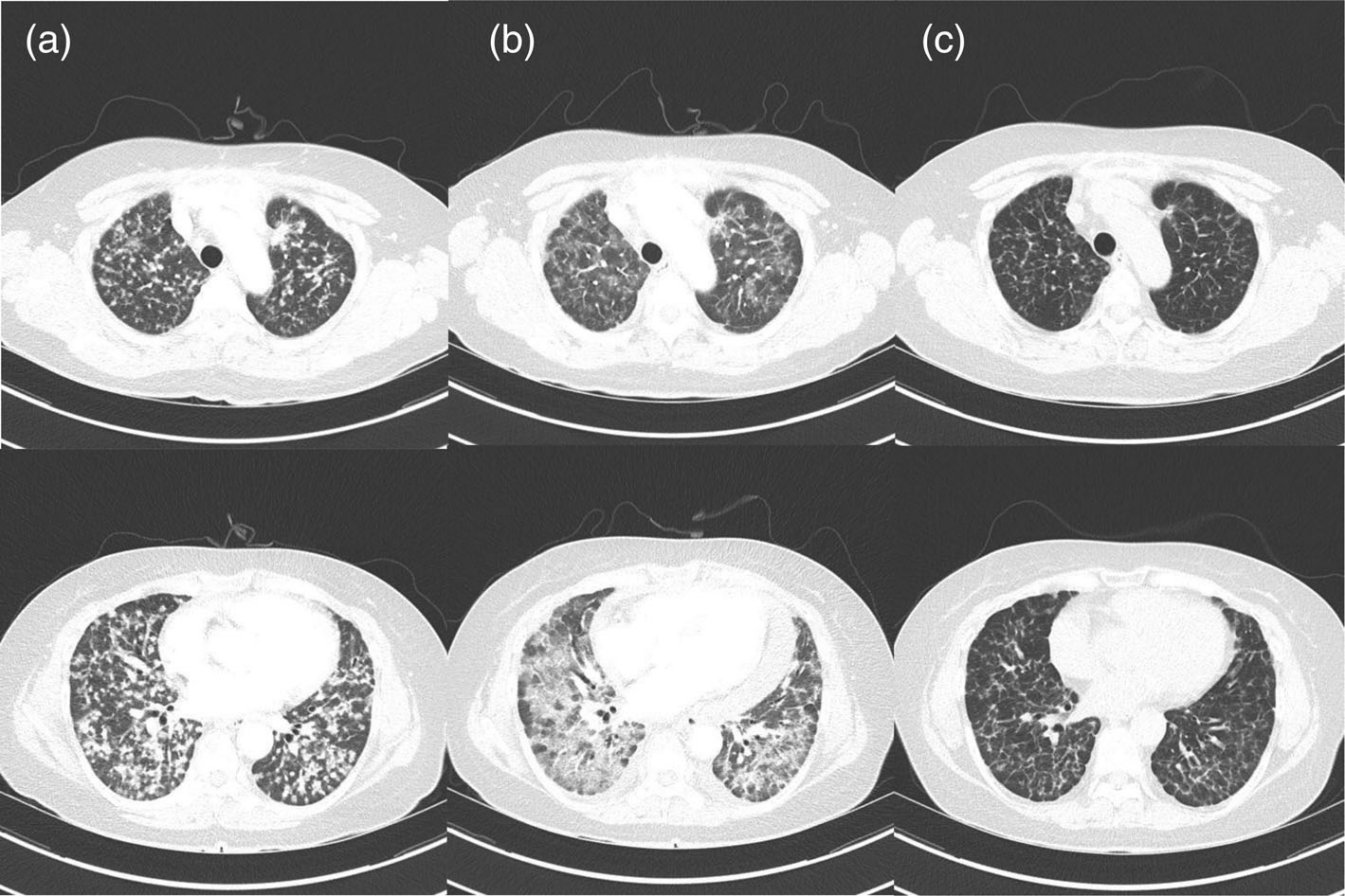
(**a**) Chest computed tomography (CT) scan showed multiple small well‐defined nodules in both lungs prior to administration of osimertinib. (**b**) Six days after administration of osimertinib, CT showed marked resolution of multiple tumor nodules in both lungs, but newly developed ill‐defined areas of patchy and extensive ground‐glass attenuation in the whole lung fields. (**c**) Four months after pneumonitis, CT showed partial remission of lung cancer with no evidence of recurrent pneumonitis.

**Figure 2:**
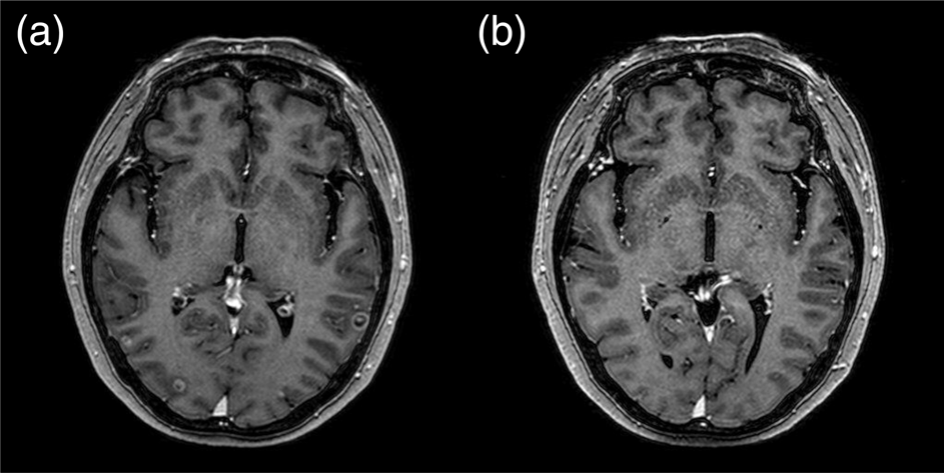
(**a**) Brain magnetic resonance imaging (MRI) revealed multiple brain metastases prior to treatment; and (b) the metastases almost completely disappeared following administration of osimertinib.

A 56‐year‐old never‐smoking woman presented to our outpatient clinic in December 2019 with suspected lung cancer. Chest computed tomography (CT) showed multiple small well‐defined nodules in both lungs (Fig 1a). Radial probe endobronchial ultrasound‐guided transbronchial lung biopsy was performed, and a diagnosis of lung adenocarcinoma stage IVb (cT4N3M1c) was made. The tumor harbored a common EGFR mutation (exon 19 deletion), and ALK and ROS1 were negative. It was accompanied by multiple brain metastases (Fig 2a), and osimertinib was prescribed at a dose of 80 mg/day orally.

After six days of treatment, the patient visited the emergency department with rapid aggravation of dyspnea. Peripheral oxygen saturation was 84% in room air. Vital signs revealed a heart rate of 110 beats per minute, blood pressure 120/80 mmHg, respiration rate 22 per minute, and body temperature 37.1°C. Laboratory tests revealed elevated lactate dehydrogenase (1138 IU/L, reference range < 550 IU/L), and C‐reactive protein (5.9 mg/dL, reference range < 0.06 mg/dL). Chest CT showed marked resolution of multiple tumor nodules in both lungs, but newly developed ill‐defined areas of patchy and extensive ground glass attenuation with inter‐ and intralobular septal thickening in the whole lung fields (Fig 1b).

Oxygen was administered intranasally to relieve the symptoms. Studies for pathogen identification, including sputum culture, streptococcal pneumonia antigen, immunoglobulin M mycoplasma antibody, whole‐blood culture, viral studies (FilmArray respiratory panel), and fungal studies, were all negative. No other causes of pneumonitis were identified.

Osimertinib‐induced pneumonitis, grade 3, was confirmed and was subsequently discontinued. Methylprednisolone was then administered at a dose of 0.5 mg/kg. Serial chest X‐rays were performed, and after 12 days, revealed improvement of the areas of patchy and extensive ground‐glass attenuation, and intranasal oxygen was discontinued. Despite advice regarding the risk of reaggravation of the pneumonitis, the patient again chose to take osimertinib. We subsequently prescribed osimertinib at an initial dose of 40 mg/day for three days, and the dose was then increased to 80 mg/day. Methylprednisolone was tapered over a period of two months and finally stopped. Currently (as of May 2020), four months after pneumonitis, the patient continues to have osimertinib 80 mg daily, and is showing good tolerance without any evidence of pneumonitis. Chest CT revealed partial remission of lung cancer with no evidence of recurrent pneumonitis (Fig 1c), and brain magnetic resonance imaging (MRI) showed that the brain metastatic lesions had almost completed disappeared (Fig 2b).

As this study was a clinical case report, no ethical committee approval was required, which is in compliance with the institutional and national policies concerning research approvals. The patient was informed that clinical details and images concerning the case would be submitted for publication, and consent was provided.

## Discussion

The third‐generation EGFR TKI osimertinib has shown greater efficacy than cytotoxic chemotherapy in patients with T790M‐positive advanced NSCLC.^3^ More recently, osimertinib has been reported to show superior efficacy to standard EGFR‐TKIs (gefitinib or erlotinib) in the first‐line treatment of EGFR mutation‐positive advanced NSCLC.^1^ In addition, osimertinib has shown prolonged median central nervous system (CNS) progression‐free survival in patients with CNS metastasis compared to standard EGFR‐TKIs.^4^

Osimertinib‐induced pneumonitis has been reported to occur at a rate of 2%–4% in clinical trials. Transient asymptomatic pulmonary opacities (TAPO) have been observed in 20% of cases with osimertinib treatment.^5^ There have been several case reports of osimertinib‐induced pneumonitis. Most cases were mild or moderate pneumonitis, and time from the first osimertinib administration to onset of pneumonitis has been reported to range from one to eight months, until our case. In our case, pneumonitis was severe with indication for oxygen, and developed six days after the first administration of osimertinib. After the pneumonitis had improved following administration of methylprednisolone, osimertinib retreatment was attempted. Four months after pneumonitis, the patient is continuing to take osimertinib 80 mg per day, and has shown a good tolerance without any evidence of pneumonitis. We present a summary of previous reports of osimertinib‐induced pneumonitis in Table 1.

**Table 1:** Osimertinib retreatment in the literature

References	Age	Sex	Time to onset	Grade of pneumonitis (by CTCAE)	Osimertinib initial dose, mg	Osimertinib retreatment dose, mg	Corticosteroid during retreatment	Recurrence of pneumonitis
Miyauchi *et al*.^6^	75	F	64 days	Grade 2	80 mg/day	40 mg/day	Yes	No
Mamesaya *et al*.^7^	38	F	31 days	Grade 2	80 mg/day	80 mg/day	No	No
Kiriu *et al*.^8^	62	M	82 days	Grade 2	80 mg/day	40 mg/day	Yes	No
Nagasaka & Gadgee^9^	82	M	Eight months	Grade 3	80 mg/day	80 mg/every other day	Yes	No
Nagasaka & Gadgee^9^	60	M	Six weeks	Grade 3	NA	NA	Yes	No
Satoh *et al*.^10^	69	F	55 days	Grade 2	80 mg/day	40 mg/day	Yes	No
Lu & Dowell^11^	57	F	Three weeks	Grade 3	80 mg/day	80 mg/every other day	Yes	No
Present case	56	F	Six days	Grade 3	80 mg/day	40 mg/day for 3 days → 80 mg/day	Yes	No

The mechanism underlying osimertinib‐induced pneumonitis is unclear, but it has been reported that EGFR‐TKIs may increase the effects of lung damage by impairing alveolar repair mechanisms.^12^ In cases with lack of serious clinical deterioration of TAPOs, it is reasonable to continue osimertinib with regular CT follow‐up.^5^ However, there are no clear clinical guidelines in cases of symptomatic osimertinib‐induced pneumonitis. Several case reports have previously described successful retreatment with osimertinib along with systemic steroids,^6^, ^7^, ^8^, ^10^ and there have been only three reports of osimertinib retreatment in cases of severe pneumonitis.^9^, ^11^ We discontinued osimertinib and prescribed steroids, as in other cases. After the patient had recovered, osimertinib was administered at an initial dose of 40 mg/day for three days, and increased to the full dose, based on successful experience with osimertinib retreatment. Our case demonstrated that osimertinib retreatment is possible while using steroids for a sufficient period in cases of extremely early onset severe pneumonitis.

In conclusion, osimertinib can be associated with pneumonitis. This case showed extremely early onset of severe pneumonitis. In such cases, discontinuation of osimertinib as well as administration of corticosteroid, and careful reintroduction of treatment with osimertinib, may be a useful treatment option, especially in patients with brain metastasis.

## Disclosure

The authors report that there are no conflicts of interest.
